# Endocrine Mucin-Producing Sweat Gland Carcinoma, a Histological Challenge

**DOI:** 10.1155/2017/6343709

**Published:** 2017-02-19

**Authors:** Mary Anne Brett, Samih Salama, Gabriella Gohla, Salem Alowami

**Affiliations:** St. Joseph's Hospital, Hamilton Health Sciences, Hamilton, ON, Canada

## Abstract

Endocrine mucin-producing sweat gland carcinoma (EMPSGC) is a rare adnexal tumor of the skin with low-grade cytological features and neuroendocrine differentiation. It has a predilection for the skin of the eyelid, but has also been reported in the face and rarely extra-facial locations. The tumor is seen more frequently in women and on average affects the elderly. It is histologically and immunohistochemically analogous to solid papillary carcinoma of the breast/endocrine ductal carcinoma in situ with a nodular, solid, papillary, and/or cribriforming architecture, neuroendocrine differentiation, and mucin production. Since it was first described by Flieder et al. in 1997, less than 60 cases have been reported in literature. We describe the morphological and immunohistochemical features of another case with a review of the common histological differential diagnoses and emphasize the salient features that help distinguish this rare neoplasm.

## 1. Introduction

Endocrine mucin-producing sweat gland carcinoma (EMPSGC) is an uncommon low-grade adnexal carcinoma of the skin with neuroendocrine differentiation. It has a predilection for facial areas, most commonly being the eyelid [1–6], but is also known to affect the cheek and rarely extra-facial sites [1]. The tumor is seen more frequently in women and usually affects elderly patients [1–3, 6].

From our case files, we describe one referral case of EMPSGC with full immunohistochemical and special stains evaluation and the histological comparison to the common differential diagnoses.

The entity of EMPSGC was first coined in 1997 by Flieder et al. [7]. They described adnexal skin tumors that had both in situ and invasive components and which had many of the histological and immunohistochemical features seen in solid papillary carcinoma of the breast [7]. Considering the close embryological relationship between sweat and mammary glands, it is not surprising that tumors seen in one location can be analogously found in the other [7].

## 2. Case Report

One referral case of EMPSGC was found over a 19-year period (1997–2016) from our electronic case files. The clinical, surgical, and follow-up information was obtained through the referring physician. Hematoxylin and eosin stained slides were reviewed, and immunohistochemical and special stain studies were performed according to our institution's standard protocols. Positive and negative controls were evaluated and working properly.

A 73-year-old woman presented with a 4 mm pearly tan nodule on the upper right eyelid. The lesion was excised in March of 2016 with the clinical impression being that of basal cell carcinoma. The excision was complete with negative margins. The patient did not have any previous skin or adnexal tumors and there was no clinical evidence of breast carcinoma. Since the excision, the patient has been doing well without recurrence. The histological diagnosis was that of EMPSGC based on the morphological characteristics and supported by immunohistochemistry and special stains.

**Figure 1:**
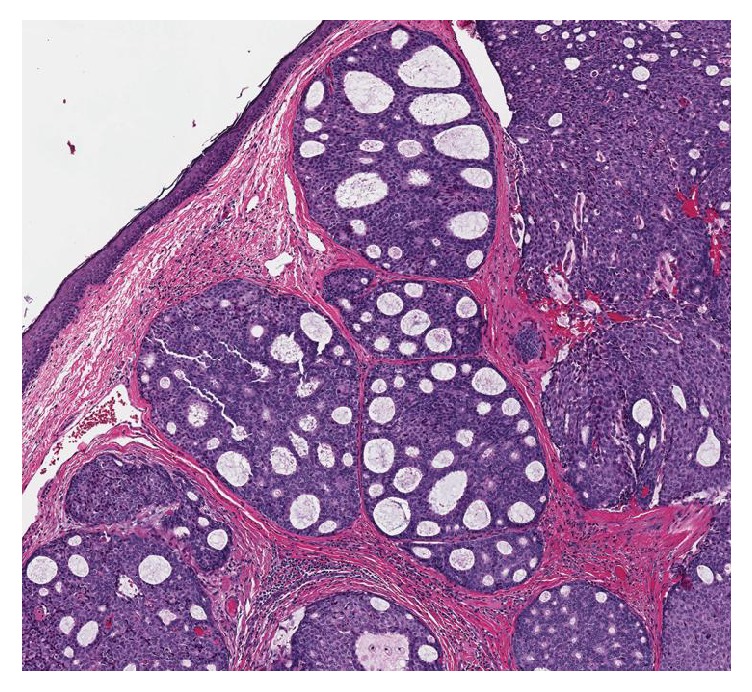
Low power H&E slide. The lobular architecture with cribriform pattern can be identified. There is no connection to the epidermis.

**Figure 2:**
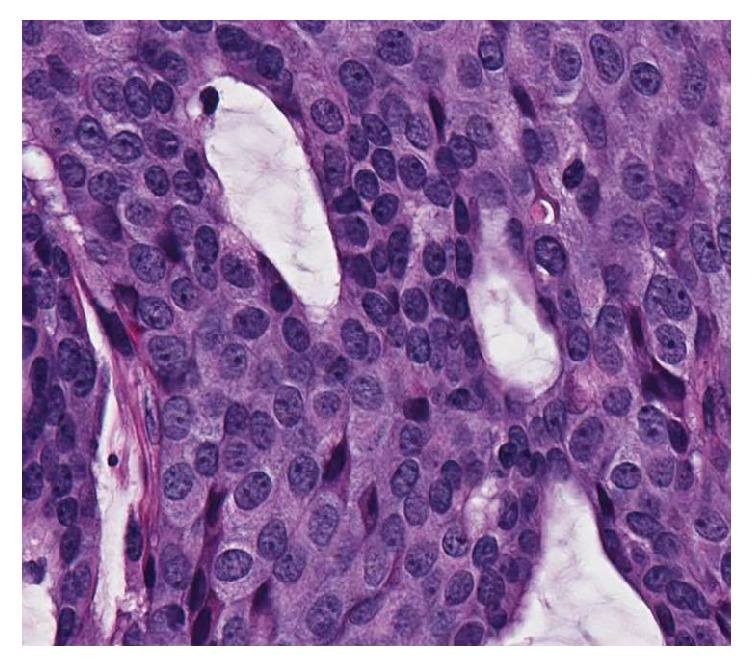
High power H&E slide. The nuclei are round to oval with conspicuous nucleoli and a finely stippled chromatin pattern.

**Figure 3:**
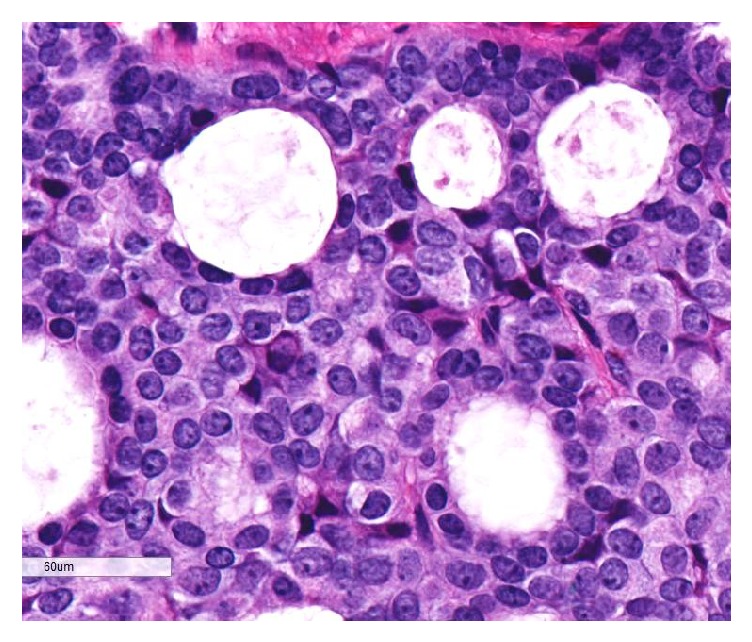
High power H&E slide. Both intracytoplasmic and extracellular mucin are present.

The tumor was dermally based, without connection to the overlying epithelium. It displayed a lobular architecture with solid and cribriforming areas (Figure 1). Within the lobules, peripheral palisading was noted around the edges, without evidence of retraction artifact. Pseudorosettes around small blood vessels in the solid areas could be identified. The nuclei were medium-sized, monomorphic, and round to oval with 1 to 2 conspicuous nucleoli, the chromatin was finely stippled and resembled the “salt and pepper” pattern seen in neuroendocrine tumors, and there was a moderate amount of eosinophilic cytoplasm (Figure 2). Intracytoplasmic mucin could be identified, as it displayed a light blue hue on H&E stained slides, and extracellular mucin was also present throughout the tumor (Figure 3). Rare mitotic figures were identified. No necrosis, nuclear pleomorphism, or lymphovascular or perineural invasion was identified.

**Figure 4:**
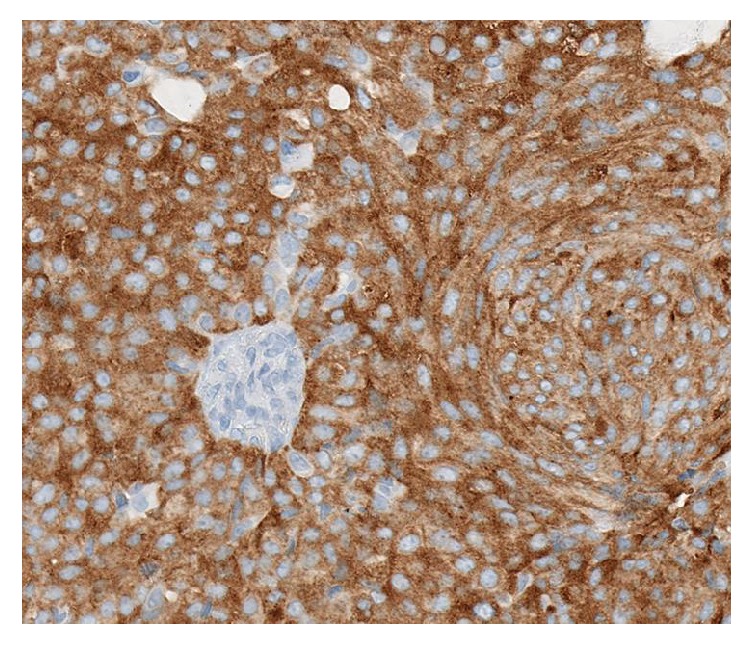
Positive synaptophysin staining.

**Figure 5:**
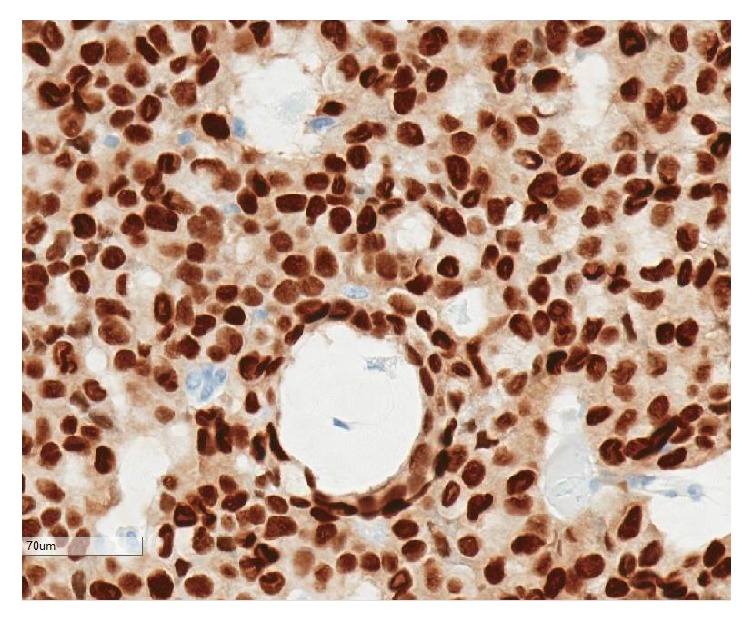
Positive estrogen receptor staining (90–100%).

**Figure 6:**
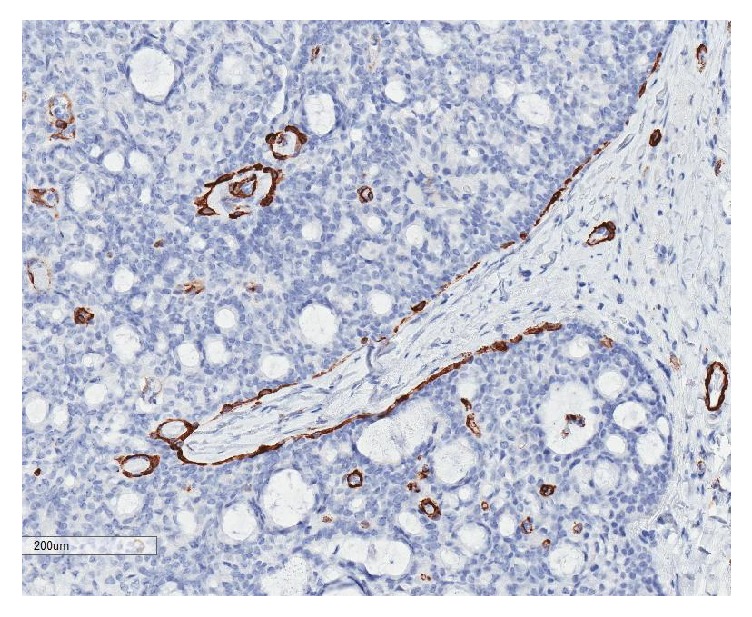
Smooth muscle myosin heavy chain (SMMHC) highlights the myoepithelial cells around the lobules.

There was strong immunohistochemical staining for synaptophysin (Figure 4), chromogranin, NSE, and CD57 and focal positive staining for CD56. Other strongly positive stains included CAM5.2, gross cystic disease fluid protein-15 (GCDFP-15), and CK7. Estrogen and progesterone nuclear stains were positive in 90–100% of the tumor cells (Figure 5). Epithelial membrane antigen (EMA) and p63 were focally positive. The Ki-67 labelling index was approximately 5%. Smooth muscle myosin heavy chain (SMMHC) stained focal myoepithelial cells around the larger lobules (Figure 6). CEA and CK20 were both completely negative. Alcian blue, mucicarmine, PAS, and PAS-D histochemistry all stained the intracytoplasmic mucin and the extracellular mucin deposits.

## 3. Discussion

EMPSGC is a rare adnexal tumor that is twice as common in females [1–3, 6] and with a predilection for the face, particularly the eyelids [1–6]. It tends to occur in individuals in their 6th and 7th decades of life [1–3, 6]. The entity was first coined by Flieder et al. in 1997 [7] and since then, less than 60 cases have been reported in the literature, making it an uncommon skin tumor that may pose diagnostic challenges to clinicians and pathologists.

Since sweat glands and mammary tissue share a common embryological origin, it is not uncommon to find analogous tumors [7]. Flieder et al. noted that EMPSGC had all the morphological and immunohistochemical features in the mammary tumor called solid papillary carcinoma or endocrine ductal carcinoma in situ [7]. These included the overall low-grade cytology, neuroendocrine differentiation, and mucin production, some of which are only identifiable by immunohistochemistry and/or special stains. In the breast, solid papillary carcinoma/endocrine ductal carcinoma in situ is deemed the precursor lesion to mucinous (colloid) carcinoma [3], and it is thought by many in the literature that this is also true in sweat glands. EMPSGC is believed to be the immediate precursor lesion to primary cutaneous mucinous (colloid) carcinoma [2, 3].

Some authors have reported focal positive myoepithelial staining with p63, SMMHC, SMA, and/or calponin in EMPSGC, while others have reported no staining whatsoever [4]. Fernandez-Flores and Cassarino proposed that EMPSGC may be best regarded as a type of pushing invasive encapsulated carcinoma, like that seen in encapsulated papillary carcinoma of the breast [4]. Encapsulated papillary carcinoma of the breast may or may not have positive myoepithelial cells, and this could explain the discrepant staining seen amongst authors for EMPSGC. Zembowicz at al., on the other hand, state that the typical loss of myoepithelial cells seen in the large nodules of EMPSGC may best be regarded as an invasive carcinoma on its own [1]. Considering loss of myoepithelial cells in the breast is regarded as evidence for invasion, perhaps this notion should be used when evaluating sweat gland tumors as well [1].

Although it would be exceedingly rare, clinicians and pathologists may want to rule out metastatic breast carcinoma to the skin before diagnosing EMPSGC [7]. Since the histological features and immunohistochemical markers are identical in both primary EMPSGC and solid papillary carcinoma of the breast, a thorough physical exam with imaging would be necessary to rule out a breast neoplasm.

Once the mass is deemed a primary skin adnexal tumor, the differential diagnosis for EMPSGC includes basal cell carcinoma (nodular subtype), nodular hidradenoma, hidradenocarcinoma, hidrocystoma, apocrine tubular adenoma, monomorphic adenoma, and dermal duct tumor [1, 2, 6, 8].

Although both EMPSGC and nodular basal cell carcinoma have a lobular architecture with uniform nuclei and cystic spaces, the latter has connections to the epidermis, retraction artifact around tumor nests, and occasionally necrosis which can give the appearance of cystic spaces. Intracytoplasmic and extracellular mucin and neuroendocrine differentiation are not seen in nodular basal cell carcinoma. Nodular hidradenomas tend to have epithelial lobules extending into the subcutaneous fat, rare tubular lumina, and multiple different cell types including round polyhedral cells, small round cells with scant cytoplasm, and clear cells. Squamous differentiation may be seen. Nodular hidradenomas also lack the uniform cells, “salt and pepper” chromatin, pseudorosette formation, and mucin production seen in EMPSGC. Hidradenocarcinoma consists of infiltrative tumor cells that display severe nuclear atypia, atypical mitoses, tumor necrosis, and lymphovascular invasion, all of which are not present in EMPSGC. Hidrocystomas show a single cystic space lined by a double layer of cuboidal epithelial cells; papillary projections into the cyst lumen and secretory tubules and ducts beneath the cyst may be seen. These tumors lack an epidermal connection, like most EMPSGC, but do not display the nodular architecture with solid and cribriform areas, “salt and pepper” chromatin, or mucin production as in EMPSGC. Apocrine tubular adenoma is a well-circumscribed dermal tumor with tubules lined by a double layer of myoepithelial cells and columnar cells with apocrine differentiation. Apocrine differentiation is characterized by round nuclei with abundant eosinophilic cytoplasm. These tumors lack a nodular architecture with solid, papillary, and cribriforming areas, neuroendocrine differentiation, pseudorosette formation around blood vessels, and mucin production. Monomorphic (canalicular) adenoma is a lobulated cystic tumor with a characteristic canalicular pattern with cords and ribbons of columnar cells displaying “beading” (columnar cells abutting each other in tubules). The nuclei are round-oval with scant eosinophilic cytoplasm and rare mitoses. Foamy histiocytes, hemorrhage, and calcifications may be identified. When comparing these tumors to EMPSGC, they lack a nodular and solid architecture, chromatin stippling, and mucin formation. Dermal duct tumors have broad anastomosing cords of columnar tumor cells with numerous tubular structures. The nuclei are monomorphic and basaloid with uniform chromatin, and clearing may be seen. These tumors form rare attachments to the epidermis. Although these tumors are monomorphic with many tubular structures as in EMPSGC, they lack a nodular, solid, and cribriforming architecture, neuroendocrine features, and mucin production. EMPSGC also lacks any attachment to the epidermis, although rare cases with such findings have been reported [9, 10].

With regard to treatment options, since this tumor tends to occur in cosmetically sensitive areas like the eyelid and cheek, skin preserving surgery is often preferred. Mohs' procedure is often the surgery of choice with an additional excision around Mohs' defect to ensure complete removal and negative surgical margins [11]. If there is a concern that the margins may be positive, then wide excision with reconstruction is recommended [11].

From published literature, the overall prognosis of EMPSGC is excellent, although there are 2 reported cases of local recurrence possibly due to the highly sensitive facial areas these tumors tend to occur leading to positive resection margins [5, 12]. There have been no reported cases of distant metastases. When EMPSGC is associated with an invasive mucinous carcinomatous component, the risk of recurrence is approximately 30% and with a higher incidence of direct extension into adjacent structures and lymph node metastases [13]. Since the average time for EMPSGC recurrence is 24 months, it is recommended that these patients undergo at least 2 years of follow-up after their resection [5, 6, 14].

## 4. Conclusion

EMPSGC is an uncommon malignant adnexal tumor which has analogous histological and immunohistochemical findings with solid papillary carcinoma of the breast [7]. It is therefore pertinent that a comprehensive physical exam with imaging is completed to rule out a primary breast lesion with metastases. When considering EMPSGC, the differential diagnosis also includes other skin tumors especially nodular basal cell carcinoma, nodular hidradenoma, hidradenocarcinoma, hidrocystoma, apocrine tubular adenoma, monomorphic adenoma, and dermal duct tumors [1, 2, 6, 8]. If a tumor has a lobular architecture with solid, papillary, and/or cribriform areas, evidence of neuroendocrine differentiation, and mucin production, EMPSGC should be considered.
